# Rare Presentation of Angiolipoma Affecting an Intact Achilles Tendon

**DOI:** 10.1155/2022/6997320

**Published:** 2022-03-07

**Authors:** Takeo Mammoto, Nobuyuki Takahashi, Norio Takayashiki

**Affiliations:** ^1^Department of Orthopaedic Surgery and Sports Medicine, Tsukuba University Hospital Mito Clinical Education and Training Center, Mito Kyodo General Hospital, University of Tsukuba, 3-2-7 Miya-Machi, Mito, Ibaraki 310-0015, Japan; ^2^Department of Radiology, Tsukuba Memorial Hospital, 1187-299, Kaname, Tsukuba, Ibaraki 300-2622, Japan; ^3^Department of Pathology, Tsukuba University Hospital Mito Clinical Education and Training Center, Mito Kyodo General Hospital, University of Tsukuba, 3-2-7 Miya-Machi, Mito, Ibaraki 310-0015, Japan

## Abstract

Musculoskeletal lipomatous lesions are common in soft tissues. However, these are rarely associated with tendon sheaths or tendon compartments. Moreover, angiolipoma of the Achilles tendon is yet to be described. Here, we report an angiolipoma of an intact Achilles tendon, which has not been described previously. A 54-year-old woman presented with a two-year history of a palpable mass in the posterior aspect of the left ankle. The mass caused an intermittent localized pain while walking and a catching phenomenon induced by the plantar dorsiflexion movement of the ankle joint. Magnetic resonance imaging revealed a well-circumscribed, oval lesion on the lateral aspect of the Achilles tendon. The location and shape of the lesion had changed over time, suggesting that the lesion was moving in and out around the Achilles tendon. At the surgery, the tumor was confirmed under the crural fascia. Histopathological examination revealed that the tumor comprised mature adipocytes covered peripherally with a fibrovascular capsule. Based on these features, the tumor was diagnosed as an angiolipoma. Angiolipomas are typically treated surgically by simple excision, and lipomatous lesions of the tendon sheath are not different. From this case report, angiolipomas are rare but should be considered in the differential diagnosis and treatment of Achilles tendon tumors.

## 1. Introduction

Musculoskeletal lipomatous lesions are common in soft tissues. These can occur anywhere on the body and are typically localized in subcutaneous fat. Angiolipoma, one of the lipomatous lesions, represents a benign subcutaneous lesion as described by Howard and Helwig [1]. This most commonly affects young male individuals, and the most frequent site of involvement is the forearm followed by the trunk and upper arm [2]. Lipomatous lesions may occasionally occur in a tendon sheath or tendon compartment and are labeled according to location [3, 4]. Lipomatous lesions of the tendon sheath are of two types: (A) focal lipomatous lesions in the affected tendon sheath, called lipoma simplex, and (B) a lipoma-like lesion comprising hypertrophic synovial villonodular proliferation distended with fat, called lipoma arborescens [2]. However, lipomatous lesions are rarely associated with tendon sheaths or tendon compartments [2–4]. Bryan et al. reviewed the literature and found 43 reported cases of lipoma of the tendon sheath [3]. Of these, 33 involved the hands or wrists, and only 10 cases involved the ankle or foot. Two of the nine cases reported a lipoma in the Achilles tendon sheath, which was originally reported by Mannini in 1928. One such report was of a lipoma simplex and the other of a lipoma arborescens. Unfortunately, these cases did not provide details regarding anatomical sites. Recently, Khan et al. reported a case of lipoma within the Achilles tendon sheath [4]. They reported that they incidentally found a lipoma upon operative repair of a ruptured Achilles tendon, which did not involve the tendon arising from within the paratenon. Here, we report an angiolipoma affecting an intact Achilles tendon, which has not yet been previously described.

## 2. Case Presentation

**Figure 1:**
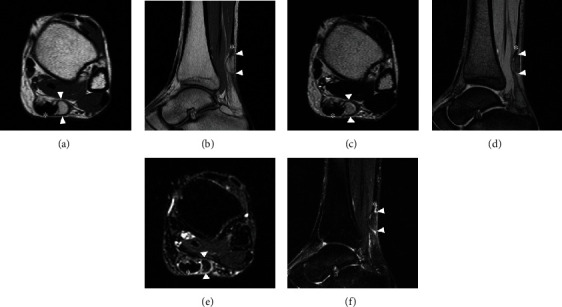
Magnetic resonance (MR) imaging at first visit. Axial (a, c, and e) and sagittal (b, d, and f) T1-weighted (T1WI, a and b), T2-weighted (T2WI, c and d), and fat-suppressed T2-weighted (T2FS, e and f) MR images.Images show a lesion (arrowhead) at the lateral aspect of the Achilles tendon (asterisk). It is isointense relative to subcutaneous fat and is surrounded by a low signal intensity area, which allows it to be distinguished from adjacent adipose tissues. The continuity of the Achilles tendon was maintained, and no tendon injury was indicated.

**Figure 2:**
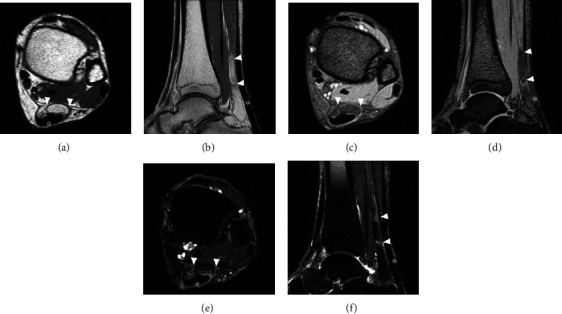
Magnetic resonance (MR) imaging at the second visit. Axial (a, c, and e) and sagittal (b, d, and f) images of T1WI, T2WI, and T2FS MR images. The second MR image shows that the lesion has migrated to the ventral aspect of the Achilles tendon. The location and shape of the lesion has changed compared with the previous findings on the previous MRI, suggesting that the lesion was moving in and out around the Achilles tendon.

**Figure 3:**
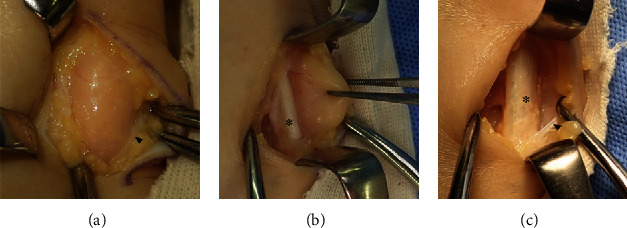
Intraoperative photograph of the lesion. The tumor is located under the crural fascia (arrowhead). The paratenon was partially continuous with the surrounding capsule of the lipoma. There was no adhesion adjoining the lesion to surrounding tissues. No significant damage or degeneration of the Achilles tendon (asterisk) was confirmed.

**Figure 4:**
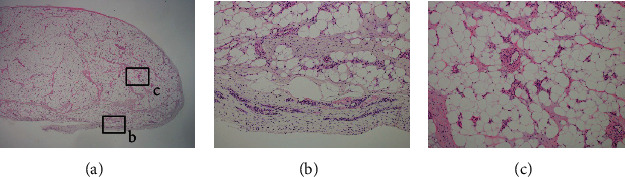
Histopathological findings of the tumor Photomicrographs (original magnification, HE stain) (a, ×40; b and c, ×400) reveal that the tumor comprises mature adipocytes covered with a fibrovascular capsule, peripherally. Blood vessels and fibrous components are observed in the septum. The blood vessels are small-caliber capillaries. No findings consistent with malignancy were found.

A 54-year-old woman with a two-year history of a palpable mass in the posterior aspect of the left ankle was referred to our hospital. The mass caused intermittent localized pain while walking, and catching phenomenon was induced by plantar dorsiflexion of the ankle joint. The patient had no history of antecedent trauma, signs of infection, or abnormal lipid metabolism. Physical examination showed an elastic, soft, and smooth lesion on the lateral aspect of the Achilles tendon. The lesion moved with dorsiflexion and plantar flexion of the ankle, suggesting an anatomical relationship with the tendon. A plain radiograph showed a soft tissue shadow without calcification on the dorsal aspect of the Achilles tendon. Magnetic resonance imaging (MRI) revealed a well-circumscribed, oval lesion on the lateral aspect of the Achilles tendon (Figure 1). The lesion was isointense relative to subcutaneous fat on T1-weighted images (T1WIs) and T2-weighted images (T2WIs). This was suppressed on fat-suppressed T2-weighted images (T2FSs). This lesion was surrounded by a low signal intensity area on T1WIs and a high signal intensity area on T2WIs; this allowed the lesion to be distinguished from adjacent adipose tissue. The continuity of the Achilles tendon was maintained, and no tendon injury was detected on MRI scans. A benign, fat-containing, and soft tissue tumor, such as a lipoma, was suspected. Although excision surgery was contemplated, the patient's subjective perception was that the lesion disappeared and she did not want to undergo excision; thus, she was temporarily lost to follow-up. Nine months later, she visited the hospital again because the tumor had recurred. This time the tumor was removed and retracted upon plantar dorsiflexion of the ankle joint. MRI showed that the lesion had migrated to the ventral aspect of the Achilles tendon (Figure 2). The location and shape of the lesion had changed compared with what was previously observed in the MRI studies, suggesting that the lesion was moving in and out around the Achilles tendon. Eventually, excision surgery was performed. At the time of surgery, the tumor was confirmed upon incision of the crural fascia (Figure 3). The tumor was located under and was partially continuous with the paratenon. There was no adhesion to the surrounding tissues, and the margins were clear. There was no significant damage or degeneration of the Achilles tendon. Histopathological examination revealed that the tumor comprised mature adipocytes covered peripherally with a fibrovascular capsule (Figure 4). The adipocytes had exhibited irregular diameters, and blood vessels and fibrous components were observed in the septum. The vessels were small-caliber capillaries. No findings consistent with malignancy were observed. Based on these features, the tumor was diagnosed as an angiolipoma. No clinical local recurrence was observed 18 months after surgery.

## 3. Discussion

An angiolipoma represents as benign subcutaneous lesion most commonly affecting young male in the second to third decades of life [2]. The most common site of involvement is the forearm, followed by the trunk and upper arm [2]. An angiolipoma of the Achilles tendon reported here has yet to be described. Lipomatous lesions of the tendon sheath, whether in the upper or lower extremities, are extremely rare. There are only a handful of case reports on a lipoma in the Achilles tendon in literature, and, to the best of our knowledge, no angiolipoma has been observed in an intact Achilles tendon. Although rare, it is important to be aware of its existence and to be familiar with the standard of treatment.

Soft tissue lesions of the Achilles tendon have been reported including chondroma, angioleiomyoma, and chondrolipoma [5–7]. MRI is a useful tool for the diagnosis of soft tissue lesions and aids in planning the operative approach [8]. In this case, the lesion was uniformly isointense relative to subcutaneous fat on T1WIs and T2WIs and suppressed on T2FSs. This suggests a benign, fat-containing, soft tissue tumor, such as an angiolipoma. Lipomatous lesions are usually benign but can transform into liposarcomas in rare cases [2, 8]. Interestingly, this lesion was surrounded by a high signal intensity area on T2WIs and T2FSs. Histopathological examination revealed a lipomatous mass, which was covered peripherally with a fibrovascular capsule. It is unclear what this indicates. However, considering that the tumor had been moving along with ankle motion, the fibrovascular capsule may suggest that the tumor encounters and exerts friction as it moves around the Achilles tendon. Khan et al. reported encountering a lipoma incidentally present within the tendon sheath upon repair for a ruptured Achilles tendon [4]. During that procedure, they hypothesized that the lipoma may not have caused any premorbid structural weakness because the lipoma did not involve the tendon itself; the history was consistent with a traumatic rupture of the Achilles tendon. That said, the presence of a lipoma in the tendon sheath might affect the structural integrity of the tendon due to irritation from the mass. In this case, the tumor existed under the crural fascia and was connected to the paratenon. The affected tendon should be inspected carefully at the time of surgery to determine whether other procedures, such as tenorrhaphy, are required. Although this has not been studied or assessed before and it is still unclear, the presence of lipomatous lesions could cause damage or degeneration of the Achilles tendon due to irritation from the mass. Angiolipomas are typically treated surgically by simple excision, and lipomatous lesions of the tendon sheath are not different. If complete excision of an angiolipoma or a tendon sheath lipomatous lesion is performed, the postoperative recurrence rate is expected to be less than 5% [3, 9]. In this case, no recurrence was observed after 18 months of follow-up; however, continuous careful observation was still required.

A tendon sheath comprises fibrous connective tissue consisting of a fibrous layer and a synovial layer. The fibrous layer supports and protects the tendon, and the synovial layer lubricates the tendons and produces synovial fluid to prevent tendons from adhering to surrounding structures and from damage that could occur with repetitive movements [10].

In contrast, the Achilles tendon is surrounded by *a paratenon* throughout its length. The paratenon forms a thin space between the Achilles tendon and crural fascia. Although the paratenon is probably not a true tendon sheath, it still functions as an elastic sleeve [10]. Whether or not the term “Achilles tendon sheath” is appropriate, an angiolipoma originating from the tissue around the Achilles tendon is extremely rare, and we believe this report is meaningful. In conclusion, angiolipoma affecting an intact Achilles tendon has just been described. From this case report, angiolipomas are very rare but should be considered in the differential diagnosis and treatment of Achilles tendon tumors. Angiolipomas are typically treated by simple surgical excision. Evaluation of tumors for surgical resection should include awareness of the surrounding tissues and a plan to manage them appropriately. This may inform the decision on whether tumor resection alone or also tenorrhaphy is required, thus altering the outcomes for the patient.

## Data Availability

The data used to support the findings of this study are included within the article.
